# Design of therapeutic siRNAs for potential application to infection with chikungunya virus

**DOI:** 10.1016/j.heliyon.2025.e41824

**Published:** 2025-01-10

**Authors:** Ahmed Ahsan Adib, Muhammad Manjurul Karim

**Affiliations:** Department of Microbiology, University of Dhaka, Dhaka, 1100, Bangladesh

**Keywords:** Chikungunya, RNA interference, Gene silencing, Therapeutics

## Abstract

Emergence of the Chikungunya virus (CHIKV) is a new threat in the world. The disastrous effect of this virus and the unavailability of specific drugs complicated the control and management of the disease. The development of a siRNA-based drug using multiple computational tools could be a way out as one of its therapeutics. Currently, very few siRNAs against CHIKV have been computationally designed and published. Here, we considered various parts of the CHIKV genome encoding different essential protein-coding genes for designing siRNAs with a view to silencing them, thereby rendering the virus inactive. Seven potential primary siRNAs were constructed, of which, five are hereafter recommended to be used as a therapeutic tool against the virus.

## 1 Introduction

Chikungunya, a disease that causes a debilitating febrile illness characterized by severe arthralgia, is caused by the Chikungunya virus (CHIKV). CHIKV is an Alphavirus, a member of the family *Togaviridae* [1], and primarily transmitted by mosquitoes, *Aedes* spp, principally by *A. furcifer*, *A. taylori*, *A. africanus* and/or *A. luteocephalus* [2]. First identified in Tanzania in 1952 [3], CHIKV has become a significant public health concern, with 3 million infections occurring annually. Although mortality rate is relatively low (1:1000), severe and long lasting arthritis-like symptoms can persist in infants and elderly [4].

CHIKV is an enveloped, positive-sense RNA virus with a positive-sense single-stranded genome of about 11.6 kb [2]. The viral genome contains two open reading frames (ORFs). The large ORF covers almost two-thirds of the genome and encodes 4 nonstructural proteins that are essential for viral reproduction such as genome replication, RNA capping, and polyprotein cleavage. The smaller ORF encodes six proteins, including three main structural proteins: capsid protein and the transmembrane glycoproteins E1 and E2. The composition of the host-cell-derived lipid bilayer strongly resembles the plasma membrane of the infected host cell. The E1 and E2 proteins are arranged in spikes and penetrate the host cell after the virus gets involved in circulation [5].

Despite the significant concern of CHIKV infections on global health, no drug to date is available for preventing Chikungunya [6], and neither there is any effective vaccine [7]. Treatment is generally focused on relieving the symptoms. Although in recent years, there have been some development in figuring out a different therapeutic approach towards the CHIKV infection. Studies have been conducted with different medicinal plant extracts and their impact on showing cytopathic effect [8] and inhibiting viral replication [[9], [10], [11], [12]], entry [11,13], and attachments [11,14]. Various research initiatives have also been taken to design inactivated [15,16], subunit [[17], [18], [19]] and live-attenuated [[20], [21], [22]] viral vaccine against CHIKV; and some of them have undergone clinical trials [23,24], but none of the designed vaccines is readily available yet.

RNA interference (RNAi) has emerged as a promising therapeutic solution for viral infections. Small interfering RNA (siRNA), which are produced synthetically using solid-phase chemical methods, are double-stranded (ds) RNA molecules, 21 nucleotides long having a 2-nucleotide long overhang at each end. These siRNA molecules form a complex with the RNA-induced silencing complex (RISC). This complex entity binds with the target nucleotide and interrupts the expression of the corresponding genes [25]. Provided that these target sequences fall into the region that is involved in producing vital proteins for viral propagation and infectivity, siRNA holds the potential to be used as a viable drug candidate. The siRNA can be delivered using transfection, electroporation, and viral gene transfer into the target cells.

To battle emerging infections and solve existing health problems, momentum is gaining toward designing a siRNA-based drug that can be specifically delivered to the target site. The use of siRNA-based drugs are currently being used in cancer therapeutics in eliminating mutations in oncogenes and tumor-suppressing genes [26,27] and several studies have been conducted where siRNA-based drugs have entered the clinical trial phase with promising prospects [28]. siRNA have been successfully reported against several viruses, such as SARS-CoV-2 [29,30], Respiratory syncytial virus (RSV) [31], Parainfluenza virus (PIV) [32] and Adenovirus [33] infections.

If siRNAs can be designed specifically to target the essential genes of the pathogens and have no off-target effects, this could be an effective treatment strategy. Here, we report on the designing of siRNAs for combating CHIKV infections.

## 2 Materials and methods

**Fig. 1:**
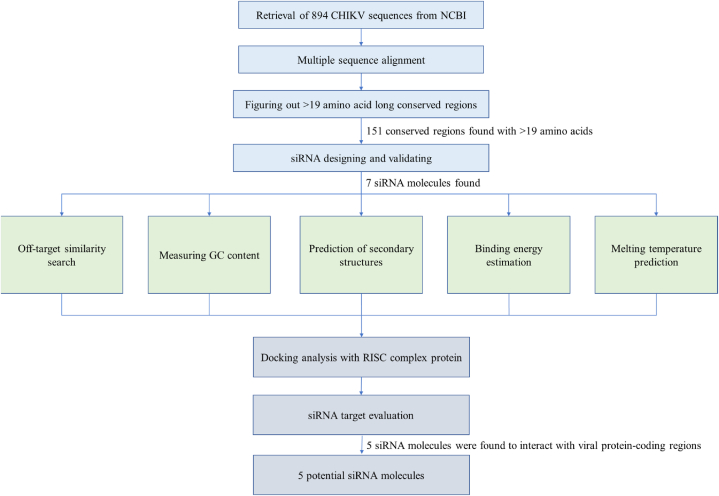
Schematic diagram of workflow to identify the potential siRNA candidates that can be used against Chikungunya virus.

We performed this study in a stepwise manner that involved retrieval of the whole genome sequences of the Chikungunya virus, designing of siRNA molecules, and in silico qualitative characterization of siRNAs to identify the specific and most promising siRNA candidates (Fig. 1).

### 2.1 Sequence retrieval and alignment

A search of the NCBI database (http://www.ncbi.nlm.nih.gov/) with ‘Chikungunya virus complete genome’ yielded 921 results. Of these, 27 were partial sequences and whole genomes with missing nucleotides, thus these were excluded from further analysis as these will hinder finding the conserved regions. The remaining 894 CHIKV whole genome sequences were retrieved in fasta format. The sequences were aligned using the MAFFT [34] version 7 (https://mafft.cbrc.jp/alignment/server/) online server with all the parameters set to their default values.

### 2.2 Filtering the conserved regions and siRNA designing

Given the large number of sequences, we opted to take the regions that are conserved >97 % of the sequences. Since a minimum sequence length of 19 nucleotides is necessary for both the siRNA guide strand and its corresponding target to initiate siRNA-medicated gene silencing, only the conserved sequences that are ≥19 nucleotides long are considered for figuring out the capability of synthesizing siRNA molecules [35]. These conserved sequences ≥19 nucleotides were listed and analyzed with siDirect2.0 (http://sidirect2.rnai.jp/) webserver to obtain potential siRNA duplexes [36,37]. The design of siRNAs followed the criteria outlined by Ui-Tei, Amarzguioui, and Reynolds rules, as described in Table 1. The melting temperature was set below 21.5 °C so that potential siRNA duplex formation can occur with minimized possibility for off-target bindings [38].

**Table 1:** Rules for designing siRNA molecules.

Rules	Description
Ui-Tei rules [39]	• A/U at the 5′ terminus of the sense strand • G/C at the 5′ terminus of the antisense strand • At least 4 A/U residues in the 5′ terminal 7 bp of sense strand • No GC stretch longer than 9 nt
Amarzguioui rules [40]	• Duplex end A/U differential >0. Strong binding of 5′ sense strand • No U at position 1. Presence of A at position 6 • Weak binding of 3′ sense strand. No G at position 19
Reynolds rules [41]	• Each rule is assigned a score which is summed up to a total duplex score to improve the efficacy of siRNA

### 2.3 Further validation of the designed siRNAs

To ensure the accuracy of the siRNA molecules designed by the siDirect2.0 webserver, further validations were performed by using the OligoWalk webserver (https://rna.urmc.rochester.edu/cgi-bin/server_exe/oligowalk/oligowalk_form.cgi) [42], which is an online tool that designs siRNA molecules based on thermodynamic stability. The target mRNA sequence was uploaded to the server to check the likelihood of the siRNA molecules to be effective. Additionally, the siRNA Scales webserver (http://gesteland.genetics.utah.edu/siRNA_scales/) was also utilized to predict the level of mRNA present in the cell after the silencing. A higher probability of efficiency and lower percentage of mRNA retaining in cell would further indicate a strong siRNA silencing effect.

### 2.4 Off-target similarity search

To assess the possibility of the siRNA binding elsewhere on the human genome and shutting down host protein production, the nucleotide BLAST (Basic Local Alignment Search Tool) was carried out. Additionally, to evaluate the possibility of the siRNA molecules binding with mouse genome in the *in vivo* experiments, the off-target was extended to the mouse genome. The NCBI blast was conducted using the ‘Human genomic plus transcript’ and ‘Mouse genomic plus transcript’ databases separately. The expected threshold value was set to 10 and the BLOSUM 62 matrix was used as the parameters.

### 2.5 Measuring the GC content

Since the higher GC-content of siRNA is inversely correlated with its activity, this study aimed to keep the estimation low between 32 % and 58 % [40]. The GC content of the siRNA sequences were computed with Endmemo, an online too available at www.endmemo.com/bio/gc.php.

### 2.6 Prediction of secondary structures

The free energy of folding for the guide strands of the siRNA molecules was determined to ensure the proper dissociation between guide and passenger strands prior to binding to the target site of mRNA. All 7 guide strands of the siRNA molecules were subjected to a web tool called MaxExpect [43,44] that predicts RNA secondary structure by maximizing the expected base-pair accuracy. All the parameters were kept at their default value. The tool is accessible at www.rna.urmc.rochester.edu/RNAstructureWeb/Servers/MaxExpect/MaxExpect.html.

### 2.7 Binding energy estimation

Binding energy serves as a critical parameter for predicting enhanced gene silencing activity exerted by the siRNA molecules. To evaluate the binding between the siRNA guide strand and its target mRNA, binding energy was calculated using the DuplexFold (www.rna.urmc.rochester.edu/RNAstructureWeb/Servers/DuplexFold/DuplexFold.html) online tool. The analysis was conducted with a maximum energy difference set at 5 % and the maximum loop size of 30 and a temperature setting of 310.15K.

### 2.8 Melting temperature prediction

The melting temperature (T_m_) of the siRNA-mRNA complexes was analyzed with the DINAMelt [45] web tool. Here, the heat capacity plot is generated by using full structures of the siRNA-mRNA complexes. Two key values were obtained: (1) T_m_ (C_p_), identified as the temperature corresponding the peak if the heat capacity (C_p_) curve; and (2) T_m_ (Conc), which represents the temperature at which the given siRNA-mRNA complex will reduce to half of its initial concentrate. These metrics provide insight into the thermal stability and the dissociation properties of the complexes.

### 2.9 Docking score calculations

Gene silencing via RNAi depends on successful docking of siRNA molecules with RISC protein. More specifically, for RNAi activity to take place, the guide strand of siRNA molecules should bind to Human Argonaute2 (Ago2) protein within the RISC complex. As piwi domain in the Ago2 structure is proven to be the suitable binding site of the siRNA molecules for the RNAi to take place [46], the efficacy of the predicted siRNA molecules was done by docking of siRNA molecules in this domain. Protein structure was retrieved from PDB server (PDB ID: 5JS1), which is an Ago2-siRNA bound complex. Bound siRNA molecule in the piwi domain of this structure was removed using PyMOL software to dock the designed siRNAs in its place. Protein molecules and ligand molecules were prepared using AutoDockTools and uploaded to HDOCK webserver [47] where protein-ligand dockings were performed and analyzed.

### 2.10 Sorting and filtering potential siRNA molecules

Following the identification of potential siRNA molecules, the final step is to determine the target sites on the CHIKV genome to asses which genes are affected from expressing and their functional significance. The target regions of the siRNA molecules were mapped onto the CHIKV genome to identify affected areas. The siRNA molecules that targeting protein-coding regions were prioritized and considered as the final molecules.

## 3 Results

### 3.1 Sequence retrieval and siRNA design

A total of 894 sequences downloaded from the NCBI database was aligned to identify highly conserved regions of at least 19 nucleotides in length. This analysis yielded 151 such sequences (Supplementary 1). These sequences were uploaded onto the siDirect2.0 webserver, which identified 7 sequences meeting its design parameters (Table 2). Potential siRNA molecules were designed against these 7 targets which had the capability to silence the expression of these genes. Furthermore, these 7 siRNA targets are aligned with CHIKV whole genome RefSeq. The search showed that the target regions of siRNA2 and siRNA4 could not affect the genome where an essential protein is being produced for viral survival and replication. The other 5 siRNA molecules showed effective silencing of different genes listed in Table 2.

**Table 2:** siRNAs against the conserved genomic region of the Chikungunya virus.

Sl	Conserved regions	Target sequence 21 nt target + 2 nt overhang	siRNA sequences 21 nt *guide* (5′→3′) 21 nt passenger (5′→3′)	Seed-duplex stabil™(Tm)		Interrupted activity
				Guide	Passenger	
1	TCTGGAAAGATCGGGGACTTACAAG	TGGAAAGATCGGGGACTTACAAG	UGUAAGUCCCCGAUCUUUCCA GAAAGAUCGGGGACUUACAAG	21.4 °C	14.8 °C	Viral methyltransferase
2	AACGAAAGAGAGTTCGTAAACAGAAAG	ACGAAAGAGAGTTCGTAAACAGA	UGUUUACGAACUCUCUUUCGU GAAAGAGAGUUCGUAAACAGA	15.0 °C	19.1 °C	a
3	TGGGCTAAGAGCTTGGTCCCTATCCTCGAAACAGCGGGGATAAAACTAA	CTCGAAACAGCGGGGATAAAACT	UUUUAUCCCCGCUGUUUCGAG CGAAACAGCGGGGAUAAAACU	15.5 °C	19.2 °C	Peptidase C9
4	GAATCGGAAGAATAAGAAGCAAAAGCAAA	TCGGAAGAATAAGAAGCAAAAGC	UUUUGCUUCUUAUUCUUCCGA GGAAGAAUAAGAAGCAAAAGC	18.6 °C	14.8 °C	a
5	ATGTGCATGAAAATCGAAAATGATTG	GTGCATGAAAATCGAAAATGATT	UCAUUUUCGAUUUUCAUGCAC GCAUGAAAAUCGAAAAUGAUU	7.4 °C	13.8 °C	Peptidase S3
6	GCGCAGATACCCGTGCACATGAAGTCCGACGCTTCGAAGTTCACCCATGAGAAACC	TCGAAGTTCACCCATGAGAAACC	UUUCUCAUGGGUGAACUUCGA GAAGUUCACCCAUGAGAAACC	20.4 °C	19.2 °C	Peptidase S3
7	GTGGAGAAGTCCGAATCATGCAAAACAGAATTTGCATCAGC	TCCGAATCATGCAAAACAGAATT	UUCUGUUUUGCAUGAUUCGGA CGAAUCAUGCAAAACAGAAUU	19.2 °C	16.2 °C	Alpha E1 Glycoprotein

a In addition to target protein inactivation, the whole genome sequences were considered for siRNA designing; these two molecules are offered as examples.

The OligoWalk server analysis indicated all the predicted regions exhibiting high efficiency in silencing activity. All seven predicted siRNA molecules demonstrated greater than 0.75 of probability of efficiency, while four of them predicted to be effective >0.9. Furthermore, predictions from the siRNA Scale server estimated that the percentage of remaining mRNA molecules after the cleaving process is below 15 % for all the siRNA molecules. These results are listed below in Table 3.

**Table 3:** Evaluation of efficacy of designed siRNA molecules.

Sl	Target sequence 21 nt *target +* 2 nt *overhang*	RNA oligo sequences 21 nt *guide (5′→3′)* 21 nt *passenger (5′→3′)*	Probability of being efficient siRNA	Percentage of mRNA remained in cells after siRNA directed cleavage
1	TGGAAAGATCGGGGACTTACAAG	UGUAAGUCCCCGAUCUUUCCA <p class="">GAAAGAUCGGGGACUUACAAG	0.888344	13
2	ACGAAAGAGAGTTCGTAAACAGA	UGUUUACGAACUCUCUUUCGU <p class="">GAAAGAGAGUUCGUAAACAGA	0.948422	11
3	CTCGAAACAGCGGGGATAAAACT	UUUUAUCCCCGCUGUUUCGAG <p class="">CGAAACAGCGGGGAUAAAACU	0.865812	12
4	TCGGAAGAATAAGAAGCAAAAGC	UUUUGCUUCUUAUUCUUCCGA <p class="">GGAAGAAUAAGAAGCAAAAGC	0.9581	4
5	GTGCATGAAAATCGAAAATGATT	UCAUUUUCGAUUUUCAUGCAC <p class="">GCAUGAAAAUCGAAAAUGAUU	0.93571	13
6	TCGAAGTTCACCCATGAGAAACC	UUUCUCAUGGGUGAACUUCGA <p class="">GAAGUUCACCCAUGAGAAACC	0.933436	4
7	TCCGAATCATGCAAAACAGAATT	UUCUGUUUUGCAUGAUUCGGA <p class="">CGAAUCAUGCAAAACAGAAUU	0.755575	11

### 3.2 Off-target binding search

Predicting the off-target similarity is vital to ensure that the designed siRNA molecules do not interfere with the host's housekeeping genes essential for protein production [48]. To figure out the off-target effects, the guide strands from all seven siRNA molecules were uploaded on the blastn server. The analysis revealed no off-target binding possibilities in both human and mouse genome and transcriptome.

### 3.3 GC content calculation and structure prediction

**Fig. 2:**
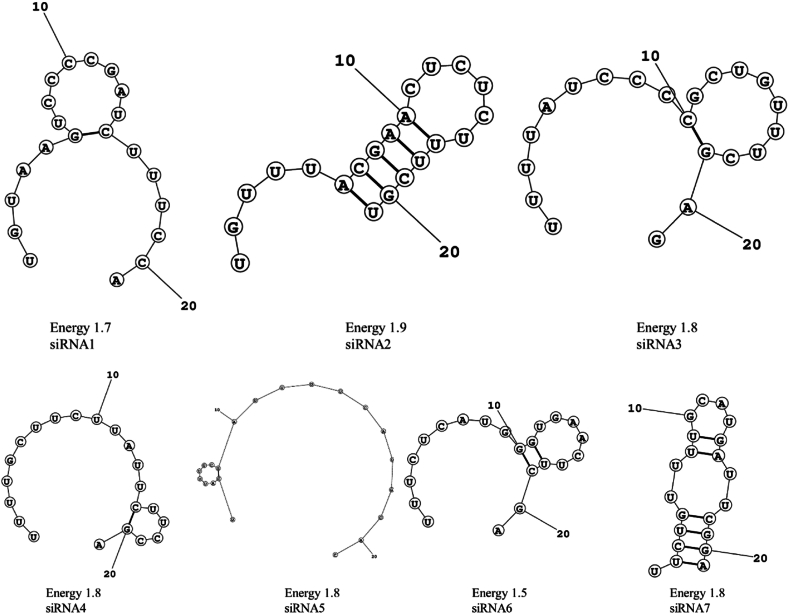
siRNAs with predicted secondary structures with energy scores *(Digits indicate the position of the nucleotide)*.

GC content of the siRNA molecules was calculated and found to range from 33.3 % to 47.6 %, which falls within the recommended range. All seven siRNA molecules were proved to contain moderate GC content, minimizing the risk of off-target effects (Table 2). Secondary structures analysis revealed that all seven molecules exhibited are thermodynamic stability to exert the effects, as indicated by energy score from the MaxExpect webserver ranging from 1.5 to 1.9 (Fig. 2 and Table 4).

**Table 4:** Features of potential siRNA molecules.

Sl	Name	Target sequence 21 nt target + 2 nt *overhang*	siRNA sequences 21 nt *guide* (5′→3′) 21 nt *passenger (5′→3′)*	GC%	Free energy of folding	Binding energy (siRNA-mRNA) kcal/mol	Tm (Cp) °C	Tm (Conc) °C
1	siRNA1	TGGAAAGATCGGGGACTTACAAG	UGUAAGUCCCCGAUCUUUCCA GAAAGAUCGGGGACUUACAAG	47.62	1.7	−38.8	91.6	90.6
2	siRNA2	ACGAAAGAGAGTTCGTAAACAGA	UGUUUACGAACUCUCUUUCGU GAAAGAGAGUUCGUAAACAGA	38.10	1.9	−33.1	84.8	83.7
3	siRNA3	CTCGAAACAGCGGGGATAAAACT	UUUUAUCCCCGCUGUUUCGAG CGAAACAGCGGGGAUAAAACU	47.62	1.8	−36.6	94.6	93.2
4	siRNA4	TCGGAAGAATAAGAAGCAAAAGC	UUUUGCUUCUUAUUCUUCCGA GGAAGAAUAAGAAGCAAAAGC	33.33	1.8	−31.5	85.6	84.1
5	siRNA5	GTGCATGAAAATCGAAAATGATT	UCAUUUUCGAUUUUCAUGCAC GCAUGAAAAUCGAAAAUGAUU	33.33	1.8	−30.7	80.2	78.9
6	siRNA6	TCGAAGTTCACCCATGAGAAACC	UUUCUCAUGGGUGAACUUCGA GAAGUUCACCCAUGAGAAACC	42.86	1.5	−36.0	90.4	89.2
7	siRNA7	TCCGAATCATGCAAAACAGAATT	UUCUGUUUUGCAUGAUUCGGA CGAAUCAUGCAAAACAGAAUU	38.10	1.8	−33.6	86.3	84.9

### 3.4 Evaluation of siRNA-target site binding ability

**Fig. 3:**
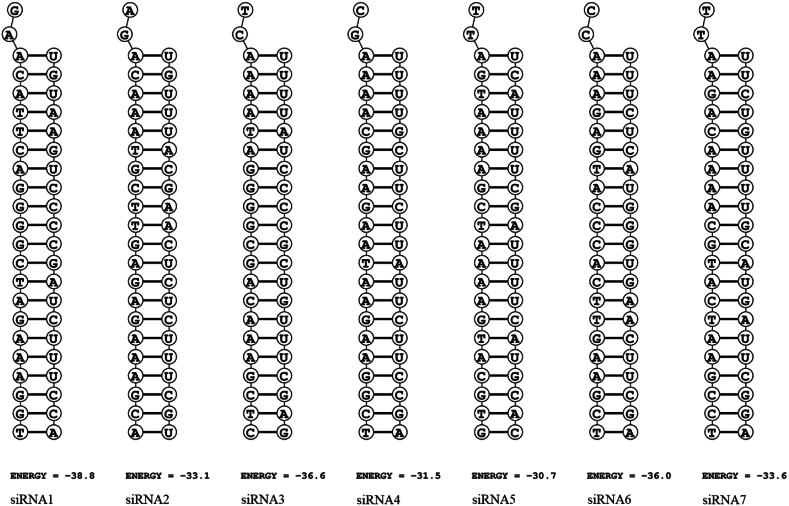
Predicted siRNA-mRNA binding (*Left strand: siRNA passenger strand; Right strand: Viral mRNA)*.

To evaluate the siRNA-mRNA binding, the ΔG value was calculated for each pair. All siRNA molecules showed strong binding affinity with their target mRNA sequences, with ΔG values ranging from −30.7 kcal/mol to −38.8 kcal/mol (Fig. 3 and Table 4).

### 3.5 Melting temperatures calculation

The high melting temperatures observed for both Tm(Cp) and Tm(Conc) suggest that the potential siRNA molecules are highly effective in binding affinity and stability. The T_m_(C_p_) value ranged from 80.2 °C to 94.6 °C while the T_m_(Conc) value ranged from 78.9 °C to 93.2 °C. The minimal difference between Tm(Cp) and Tm(Conc) further supports their stability and potential efficacy (Table 4).

### 3.6 Docking results evaluation

**Fig. 4:**
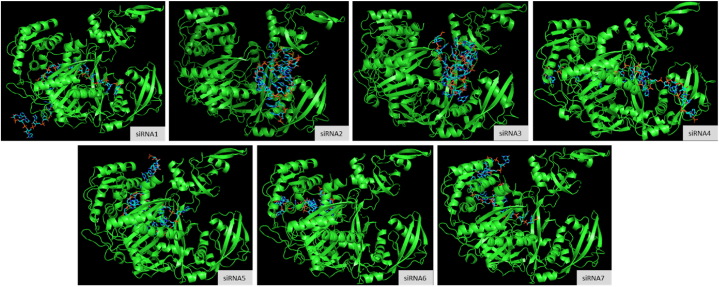
Docking state between siRNA molecules and Ago2 protein *(Green color ribbon structure represents the protein while stick structure indicates the siRNA molecules)*.

All guide strands of siRNA molecules showed strong binding with Ago2 protein situated within piwi domain of the RISC complex. The docking score ranged between −333.22 and −280.93 (Table 5), which indicates a strong binding between the protein-ligand complex. The high confidence score for the siRNA-protein binding shows a high probability of the docking to take place (Fig. 4).

**Table 5:** Docking results of siRNA - Ago2 complex.

	siRNA1	siRNA2	siRNA3	siRNA4	siRNA5	siRNA6	siRNA7
**Docking Score**	−333.22	−283.60	−305.01	−309.48	−299.79	−280.93	−302.28
**Confidence Score**	0.9750	0.9354	0.9569	0.9604	0.9524	0.9320	0.9546
**Ligand rmsd (Å)**	117.24	90.24	87.47	61.26	90.37	108.22	96.20

Of the seven siRNA molecules designed, five targeted essential CHIKV proteins and demonstrated significant silencing activity (Table 2). Among these five, three targeted structural protein formation of the virus while the other two inhibited non-structural protein production (Table 6).

**Table 6:** Potential siRNA molecules and corresponding interrupted protein.

Sl	Name	Target sequence	Protein	Protein type
1	siRNA1	TGGAAAGATCGGGGACTTACAAG	Viral methyltransferase	Non-structural
2	siRNA3	CTCGAAACAGCGGGGATAAAACT	Peptidase C9	Non-structural
3	siRNA5	GTGCATGAAAATCGAAAATGATT	Peptidase S3	Structural
4	siRNA6	TCGAAGTTCACCCATGAGAAACC	Peptidase S3	Structural
5	siRNA7	TCCGAATCATGCAAAACAGAATT	Alpha E1 Glycoprotein	Structural

## 4 Discussion

siRNA-based therapeutics have demonstrated significant potential for treating a wide range of diseases, including viral and non-viral diseases, such as cancer, dominant genetic disorders, and autoimmune disorders. This innovative technology has attracted researchers, academia, and pharmaceutical industries towards designing and development of highly effective and targeted disease therapy. For example, effective and potential siRNAs were designed, delivered and their efficacy with toxic effects and immunogenic responses were tested against MERS-CoV [[49], [50], [51]]. The work of Sohrab et al. documented observation of designed siRNAs on HEK-293 cell line against MERS-CoV, where 5 out of 10 initial siRNA molecules showed diminishing of initial viral load [50]. Given the emergence and reemergence of the CHIKV worldwide, especially in Africa [2,52] and Asia [53,54], the sporadic outbreak of the virus has been documented in regular intervals, hence became a great public health concern nowadays. This study aims to design potential siRNA molecules that would inactivate Chikungunya virus.

Recent studies have investigated the potential of siRNAs designed against E2 and nsP1 proteins of CHIKV. While 2 out of 8 designed molecules showed efficacy against the viral replication [55], the possibility of designing siRNA molecules against other proteins of CHIKV has not been fully explored. Dash et al. demonstrated efficacy of siRNA molecules targeting nsP3 and E1 genes in reducing viral replication load *in vitro* [56]. Although they showed significant reduction of viral replication activity within 24 h, persistent reduction was not observed at 72 h [56]. Here, we have attempted to design comprehensive set of siRNA molecules targeting various regions of CHIKV genome and have the capability of reducing viral replication individually or in combination.

After retrieving all 921 available CHIKV whole genome sequences from NCBI, 27 were omitted due to the presence of large gaps in the sequence. Alignment of the remaining 894 sequences resulted in 151 conserved 19-mer sequences, of which seven regions proved to have the ability to be designed for siRNA silencing. Corresponding siRNA molecules were designed (siRNA1 - siRNA7) against these seven regions maintaining the essential criteria to exert silencing activity. These siRNA molecules were further uploaded into other webservers where their validity was checked again. While siRNA7 had a moderate probability of 0.75, the remaining six molecules showed a very high probability of efficacy, ranging from 0.87 to 0.96. Additionally, these siRNAs were also predicted to efficiently cleave target mRNA, with an estimated efficiency of 87%–96 %.

These seven siRNA molecules were evaluated for off-site binding, GC content and thermodynamic stability. The predicted binding energies indicated favorable interactions between the siRNA and target mRNA, and their high melting temperature suggested that the siRNA-mRNA duplex formation would remain stable at physiological conditions.

To assess the potential for efficient RNAi, binding affinity of siRNA molecules to human Ago2 protein was evaluated using docking studies. All seven siRNA molecules have shown the capacity to strongly bind to Ago2 protein's piwi domain, as Ago2 is an important protein in the RISC complex and piwi domain is the siRNA binding region in the Ago2 structure. Finally, based on the location of the conserved regions that are targeted by the siRNA molecules, five such regions were identified that fall into the gene of several important proteins. These five siRNAs were finally selected as the designed siRNA molecules.

Among the designed siRNA molecules, siRNA1 interacts viral methyltransferase gene, which is responsible for mRNA capping. The methylation of the 5’ cap of viral mRNA is done by the viral methyltransferase enzyme [57]. This capping is a necessary step for replication in all alphaviruses, including CHIKV [58]. Halting the viral methyltransferase enzyme with siRNA1 will ensure disruption of viral replication cycle.

CHIKV, a member of Togaviridae family, contain both structural and non-structural peptidases. The non-structural peptidase C9 is involved in cleaving the large polyprotein and producing the active RNA polymerase enzyme [59]. Structural peptidase S3 is involved in viral maturation, participating in preparation of the nascent viral particles. siRNA3, siRNA5 and siRNA6, which target the structural and non-structural peptidase enzymes, will have the ability to disrupt the formation of infectious viral particles.

siRNA7 was designed to target Alpha E1 Glycoprotein. As a class II fusion protein, E1 glycoprotein trimerizes and acts as a tunnel that facilitates the entrance of viral nucleocapsid into the cytoplasm [[60], [61], [62]]. By disrupting the viral entry into the host cell, siRNA7 holds the potential to successfully inhibit subsequent viral replication.

In conclusion, five potential siRNA molecules (siRNA1, siRNA3, siRNA5, siRNA6, and siRNA7) are hereafter proposed to inactivate CHIKV as they have specific target gene sequences, while the other two, siRNA2 and siRNA4 are in the second line of choices as they attach genomic regions not involved in the protein production. This study was limited to designing the hypothetical modeling of the siRNA molecules, which must be proven by the *in vivo* experiments. Future efforts are therefore expected to test their efficacy in wet lab experiments.

## CRediT authorship contribution statement

**Ahmed Ahsan Adib:** Writing – original draft, Visualization, Validation, Software, Methodology, Data curation. **Muhammad Manjurul Karim:** Writing – review & editing, Validation, Supervision, Investigation, Formal analysis, Conceptualization.

## Data availability statement

Data will be made available on request. For requesting data, please write to the corresponding author.

## Funding source

This research did not receive any specific grant from funding agencies in the public, commercial, or not-for-profit sectors.

## Declaration of competing interest

The authors declare that they have no known competing financial interests or personal relationships that could have appeared to influence the work reported in this paper.
